# Identification and Validation of Reference Genes for RT-qPCR Analysis in Reed Canary Grass during Abiotic Stress

**DOI:** 10.3390/genes14091790

**Published:** 2023-09-12

**Authors:** Xuejie Jia, Yi Xiong, Yanli Xiong, Daxu Li, Qinqin Yu, Xiong Lei, Minghong You, Shiqie Bai, Jianbo Zhang, Xiao Ma

**Affiliations:** 1 College of Grassland Science and Technology, Sichuan Agricultural University, Chengdu 611130, China; jiaxuejie2023@163.com (X.J.);; 2 Sichuan Academy of Grassland Science, Chengdu 610097, China; ldx136@163.com (D.L.);; 3 School of Life Science and Engineering, Southwest University of Science and Technology, Mianyang 621002, China

**Keywords:** reference genes, *Phalaris arundinacea* L., abiotic stresses, real-time quantitative PCR

## Abstract

Reed canary grass (*Phalaris arundinacea* L.) is known for its tolerance to drought, heavy metals, and waterlogging, making it a popular choice for forage production and wetland restoration in the Qinghai-Tibet Plateau (QTP). To accurately assess gene expression in reed canary grass under different abiotic stresses, suitable reference genes need to be identified and validated. Thirteen candidate reference gene sequences were selected and screened using RT-qPCR to detect their expression levels in reed canary grass leaves under drought, salt, cadmium, and waterlogging stresses. Four algorithms were used to assess the stability of the expression levels of the candidate reference genes. The most stably expressed genes were *UBC* and *H3* under drought Cd, *ETF* and *CYT* under salt stress, and *ETF* and *TUB* under waterlogging stress. *GAPDH* was found to be less stable under abiotic stresses. *PIP-1*, *PAL*, *NAC 90*, and *WRKY 72A* were selected as response genes for quantitative expression assessment under drought, salt, Cd, and waterlogging stresses to confirm the accuracy of the selected stable reference genes. These results provide a theoretical reference for assessing gene expression in reed canary grass under abiotic stresses.

## 1. Introduction

Reed canary grass (RCG, *P. arundinacea* L.) is a cool-season perennial grass valued for its use in forage, bioenergy, and wetland restoration [1]. There is a wide distribution across the temperate areas of North America, Europe, and Asia [2]. Due to its rapid growth, high yield, and tall height, Reed canary grass is an exceptional forage species, serving as a vital source of hay and silage on the Qinghai-Tibet Plateau (QTP) [3]. Additionally, reed canary grass is often used to filter pollutants from aquatic environments due to its tolerance to waterlogging. It can absorb pollutants such as Al^3+^ and is a popular choice for wetland restoration [4,5]. As an energy plant, reed canary grass provides clean energy and is drought-tolerant compared to most annual plants. It requires less tillage and maintenance, and its deep-rooted nature improves nutrient use efficiency [6]. However, drought, soil salinity, heavy metals, and waterlogging are among the abiotic stresses that can limit its growth and development. Studying the transcriptional response of plants during abiotic stress can help identify genes important for critical plant pathways, leading to the development of better and more resilient canary grass varieties [7,8,9]. The study of reed canary grass is crucial for understanding its resistance to abiotic stress and gene exploitation. However, limited genomic sequence information and the lack of stable reference genes in reed canary grass hinder gene mining, gene function validation, and gene expression analysis. This lack of precision in key gene expression analysis limits the study of abiotic stress in reed canary grass. Therefore, it is crucial to further research and development in this field to identify stable reference genes in reed canary grass.

For quantifying transcript expression, real-time quantitative PCR (RT-qPCR) is widely used because it is highly sensitive, specific, reproducible, and high throughput [10,11]. The accuracy of RT-qPCR depends on expression levels of reference genes, and its results are affected by RNA quality, stable reference gene expression, cDNA synthesis, and other factors; therefore, to calibrate for experimental variability, stable reference genes must be selected for amplification with the target gene [12]. Stable reference genes have been reported to include cytoskeletal proteins and essential components in basic cellular biochemical pathways, such as ubiquitin-binding enzymes, 50S ribosomal protein L2, tubulin, etc. [13]. Although reference genes are generally considered to be stable under all conditions, it is important to note that their expression levels may not always remain consistent across different conditions, growth stages, and physiological states. Therefore, to improve the accuracy of RT-qPCR results, stable reference genes must be tested under different conditions and physiological states [14]. GeNorm, NormFinder, BestKeeper, and RefFinder are four common algorithms to evaluate the stability of candidate reference genes [15,16]. Reference gene screens have widely used these algorithms, such as those for maize (*Zea mays* L.) and larch (*Larix gmelinii (Rupr.*) Kuzen.) [17,18]. However, there are still many species lacking reference genes, and few studies have detailed the screening of reed canary grass reference genes under abiotic stress, which significantly affects the accuracy of reed canary grass gene expression.

This study aimed to identify and validate reference genes for stable expression under abiotic stresses in reed canary grass using transcriptome sequencing data. The stability of 13 potential reference genes was examined under different abiotic stresses, including salt, drought, cadmium (Cd), and waterlogging stresses. Additionally, the expression levels of *PIP-1*, *PAL*, *NAC 90*, and *WRKY 72A* genes were determined to validate the best candidate reference genes. Overall, selected and identified reference genes are important for understanding the molecular mechanisms underlying abiotic stress responses in reed canary grass and related species.

## 2. Materials and Methods

### 2.1. Plant Materials and Abiotic Stress Treatments

The seeds of reed canary grass (cv. Chuanxi; seeds obtained from Sichuan Agricultural University, Chengdu, China) were soaked in 10% sodium hydroxide for 5 min to improve germination, then rinsed in sterile distilled water. Finally, each pot was evenly sown in plastic pots with quartz sand. Twelve pots were planted with a pot size of 20 cm × 15 cm × 5 cm, 1.3 g of seeds per pot, followed by 1/2 times the amount of Hoagland nutrient solution. After sowing, the pots were grown in an incubator for 90 days under the following conditions: (1) day and night temperatures of 23 °C and 19 °C respectively; (2) photoperiod of 12 h; (3) relative humidity of 75%; (4) light intensity of 250 μmol (m^−2^·s^−1^). When reed canary grass reached the three-leaf stage, the following treatments were applied: 20%PEG-6000 solution with drought stress, 300 mmol·L^−1^ NaCl with salt stress, 50 μmol·L^−1^ CdCl with Cd stress, and waterlogging of the whole plant with waterlogging stress, with three replicates used for each treatment group. Then, leaves were collected at 0 h, 0.5 h, 1.5 h, 3 h, 6 h, 12 h, 24 h, 48 h and 72 h of each treatment, respectively, and the collected samples were immediately frozen in liquid nitrogen and stored in a refrigerator at −80 °C for subsequent RNA extraction.

### 2.2. RNA Extraction and Reverse Transcription

A frozen leaf of 0.1 g of reed canary grass was selected and pulverized by a high-throughput tissue grinder, and total RNA was extracted using the M5 HiPer Plant Complex Mini Kit (Beijing, China). Analyzing the RNA concentration and quality with the NanoDrop1 ND-1000 Spectrophotometer (Nano Drop Technologies, Wilmington, DE, USA) and agarose gel electrophoresis, respectively, confirmed the quality of the extracted RNA. The quality of the extracted RNA was confirmed by analyzing the concentration and quality of the RNA using a NanoDrop1 ND-1000 spectrophotometer (Nano Drop Technologies, Wilmington, DE, USA) and agarose gel electrophoresis, respectively. For further analysis, the first strand of cDNA was reverse transcribed using the ABSCRIPT III RT Master Mix for qPCR with gDNA Remover (Wuhan, China).

### 2.3. Primer Design and Validation

Based on the RNA-seq of the reed canary grass, thirteen candidate reference genes were selected which included *UBC* (Ubiquitin-conjugating enzyme E2 34); *GATP* (Glyceraldehyde-3-phosphate dehydrogenase); *ETF* (Eukaryotic translation initiation factor 4E); *TUB* (Tubulin alpha-3 chain); *CYT* (Cytochrome P450); *ADPF* (ADP-ribosylation factor 1); *H3* (Histone H3); *50Sr* (50S ribosomal protein L2); *MD* (3-isopropylmalate dehydrogenase 2); *Heats* (Heat shock 70 kDa protein); *Cul1* (Cullin-1); *TATA* (TATA-binding protein); *ACT* (Actin-7) [19,20]. The primers were then designed and screened for the selected candidate reference gene sequence (Table S1). The sequences of the selected candidate reference genes were used for the design and screening of the primers. Primer design was performed by Primer5 software, and primer synthesis was performed by You Kang Biotechnology (Chengdu, China) (Table S2). A melting curve analysis of the RT-qPCR reactions confirmed the specificity of the primers for candidate reference genes.

### 2.4. Quantitative RT-qPCR

For quantitative analysis, RT-qPCR was performed using the CFX96 RT-qPCR system (Bio-Rad, Singapore) and Power SYBR green PCR master mix (Wuhan, China). The temperature was maintained at zero degrees Celsius for better resolution, and the sample injection volume was kept at 10 µL. zero to one degree Celsius. The total reaction volumes were 10 µL containing 1 µL cDNA 0.2 µL forward primer. 0.2 µL reverse primer, 5 µL Genious 2 × SYBR Green Fast QPCR No ROX Mix (ABclonal, Wuhan, China) and 3.6 µL nuclease-free water. All reaction products were analyzed after 35 amplification cycles with the following steps: 10 min pre-denaturation at 95 °C, denaturation 15 s at 95 °C and annealing 1 min at 60 °C. In each abiotic stress sample, three biological replicates were performed, as well as three technical replicates.

### 2.5. Data Analysis

GeNorm [21], NormFinder [22], BestKeeper [23], and RefFinder (http://www.leonxie.com/referencegene.php, accessed on 17 January 2023) were used to determine the cycle threshold (Ct) for each reference gene. A Ct value must be converted into a relative quantification Q value as described above before NormFinder can analyze gene stability. This is mainly through the equation Q = 2^−∆Ct^, where ∆Ct = Ct sample − Ct min, t sample indicates the Ct value of the housekeeping gene, and Ct min indicates the lowest Ct value of the housekeeping gene under each abiotic stress. A coefficient of variation (CV) and standard deviation (SD) were calculated by BestKeeper using Ct values, and the expression stability measurement (M) values were calculated for each candidate reference gene via the GeNorm program (SD). Finally, RefFinder was used to combine the three software calculations mentioned above and to calculate a combined ranking index of each reference gene’s geometric mean and stability. Afterwards, the results of these four algorithms are correlated using the R language. Generally, low index values were considered reference genes of high stability. To determine the optimal number of reference genes, Vn/Vn + 1 is applied. In general, a value of Vn/Vn + 1 greater than 0.15 is required for the first V(n + 1) reference gene. Otherwise, no new reference genes need to be introduced.

### 2.6. Validation of Reference Genes

To validate the selected reference genes, reference genes, including the two most stable reference genes and the one least stable reference gene under each abiotic stress, were used to analyze the expression levels of target genes under four abiotic stresses. The target genes for each stress were selected as follows: the *PIP-1* (Aquaporin PIP1-1) was selected for drought stress, the *PAL* (Phenylalanine ammonia-lyase) for salt stress, the *WRKY 72A* (WRKY transcription factor 72A) for waterlogging stress and the *NAC 90* (NAC domain-containing protein 90) for cadmium stress(Tables S3 and S4) Then following by calculation using the 2^−∆∆Ct^ method [24].

## 3. Results

### 3.1. The Effectiveness of the Primers of 13 Specific Reference Genes

**Figure 1:**
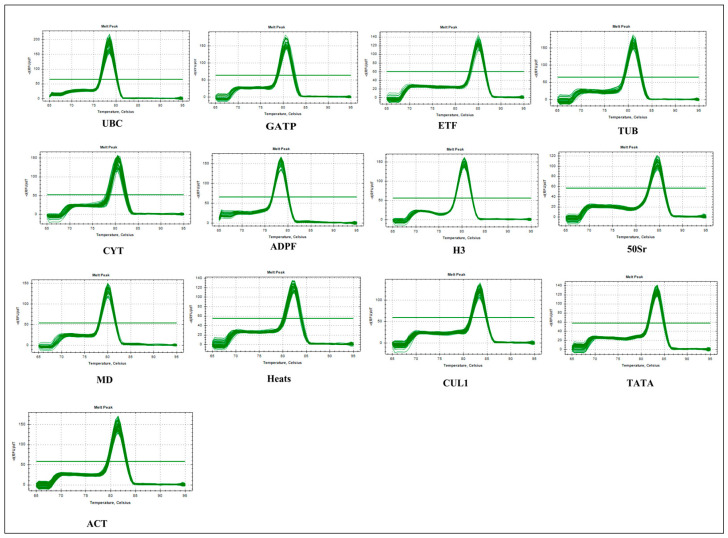
Reed canary grass melt curves for 13 candidate reference genes.

The cDNA, reverse transcribed from the total RNA of reed canary grass leaves after exposure to drought, salt, Cd, and waterlogging stresses, was used as the reaction sample for the RT-qPCR reaction. The results showed a clear single peak in the solution curve for all reference genes, indicating that the primers selected had excellent specificity and amplified the target product of each gene generated (Figure 1). Additionally, the RT-qPCR results were reproducible, and no primer dimers were produced. These findings demonstrate that the RT-qPCR results are authentic and reliable, making them suitable for use in subsequent studies.

### 3.2. Expression of Reference Gene

**Figure 2:**
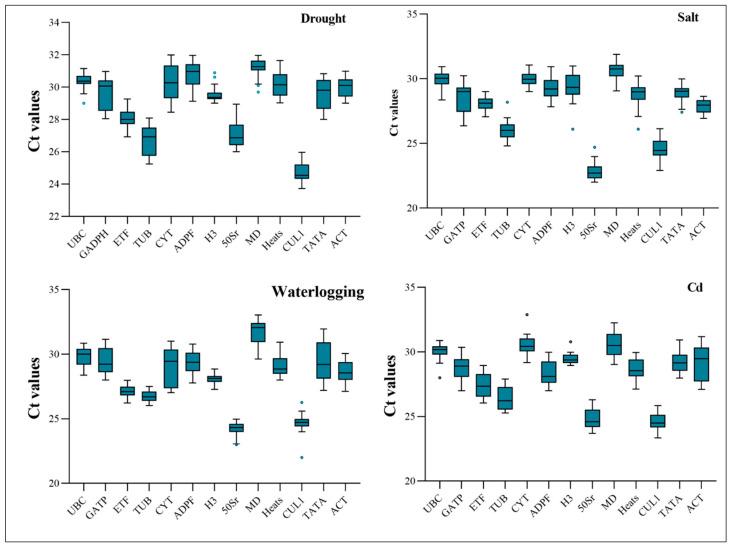
Ct values of 13 candidate reference genes in reed canary grass leaves under abiotic stresses. The variation is displayed as medians values (lines across the box plot) and the circles indicates free value.

Ct values for reference genes are inversely related to target gene expression levels. Therefore, higher Ct values indicate a lower gene expression level in the sample. Ct values for different candidate reference genes were represented by box plots. The upper and lower box plot limits indicate the maximum and minimum Ct values, and the dispersion indicates gene stability. It is more likely that a sample with a lower dispersion has more stable gene expression. A variety of abiotic stresses led to Ct values ranging from 22.92 to 32.95 for the candidate reference genes. The *UBC* gene showed the highest expression abundance under drought stress, the *CYT* gene under salt stress, the *ETF* gene under waterlogging stress and the *H3* gene under cadmium stress (Figure 2).

### 3.3. Candidate Reference Gene Stability Analysis

#### 3.3.1. GeNorm Analysis

**Figure 3:**
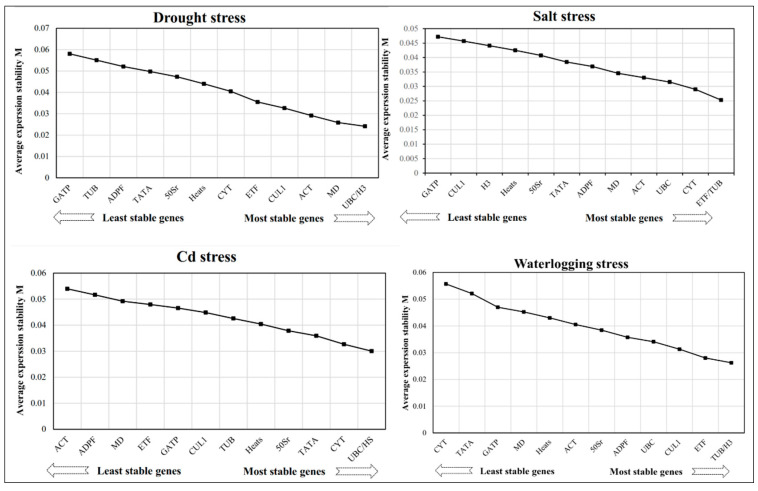
Abiotic stress effects on the expression of 13 candidate reference genes in reed canary grass leaves.

GeNorm software was used to calculate the M value, which represents the stability of the expression of the candidate reference gene. Generally, the lower the M value, the more stable the candidate gene is. The M value was used to rank the stability of the candidate reference genes. This research found that *UBC* and *H3* were the most stably expressed candidate reference genes in drought and Cd stresses, with M values of 0.03 and 0.024, respectively (Figure 3). *ETF* and *TUB* ranked highest in stability in salt stress, with M values of 0.025. In waterlogging stress, *TUB* and *H3* genes were the most stably expressed genes, with M values of 0.026. However, *CYT* was the least stable gene, with an M value of 0.057 (Figure 3).

**Figure 4:**
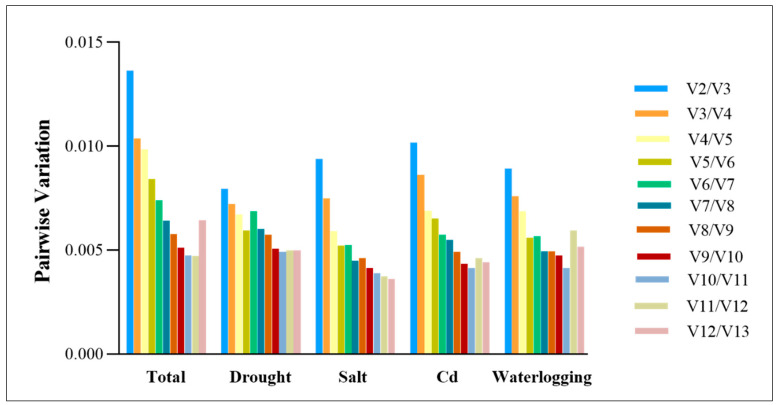
Pairwise variation (V) measures were calculated for the candidate reference genes using GeNorm.

A pairwise variation analysis can be useful for determining the optimal number of reference genes for accurate normalization. The pairwise variation values of standard factors are typically used, with a default threshold of 0.15 for V. When the Vn/Vn + 1 value is less than 0.15, n is considered the optimal number of reference genes, while n+1 reference genes are required when the value is greater. As Vn/Vn + 1 values fell below 0.15 under various abiotic stress, it was concluded that only two reference genes were required to normalize gene expression data (Figure 4).

#### 3.3.2. BestKeeper Analysis

The standard deviation (SD) and coefficient of variance (CV) of gene expression are calculated to evaluate the stability of reference gene expression. The reference gene is considered when SD is less than 1, with a higher SD indicating lower reference gene stability. In this study, *H3* and *UBC* were found to have high stability of expression under drought stress. *CYT* and *ETF* were the most consistently expressed genes under salt stress, while *H3* and *UBC* showed stable gene expression trends compared to other genes under Cd stress. *UBC* and *GATP* showed stable expression under waterlogging stress. However, it is important to note that these results are partially inconsistent with the results of the GeNorm algorithm and, therefore, require further analysis using other algorithms (Table 1).

#### 3.3.3. NormFinder Analysis

In NormFinder V20, a variance method is used to calculate the stability of candidate reference genes (S-values), with lower S-values indicating higher gene stability. The S-values of *ACT* and *UBC* under drought stress were 0.353 and 0.372, respectively, lower than those of other candidate reference genes, indicating that *ACT* and *UBC* had higher stability under drought treatment (Table 2). *ETF* and *CYT* were more stable under salt stress, with S-values of 0.362 and 0.382, respectively. *H3* and *UBC* were ranked as the top stability candidates under Cd stress, while *H3* and *ETF* were more stable than other genes under waterlogging stress (Table 2).

#### 3.3.4. RefFinder Analysis

ReferenceFinder V1.0 combines three algorithms, GeNorm, NormFinder, and BestKeeper, to rank candidate reference genes with respect to their stability. It is commonly used to analyze the combined stability of these genes. Under drought stress, *UBC* and *H3* were the most stable genes, whereas *ETF* and *CYT* were the most stable genes under salt stress. Similarly, the best reference gene combinations were *UBC* and *H3* under Cd stress, and *ETF* and *TUB* showed higher expression stability under waterlogging stress (Table 3).

To compare the correlation between the four algorithms, we calculated the correlation coefficients of the results of the four algorithms. We found that the correlation between the four algorithms was greater than 0.75, of which the GeNorm and ReFfinder algorithms had the highest correlation, and the results of the calculated gene stability were similar (Figure S1).

### 3.4. Validation the Stability of Reference Genes

**Figure 5:**
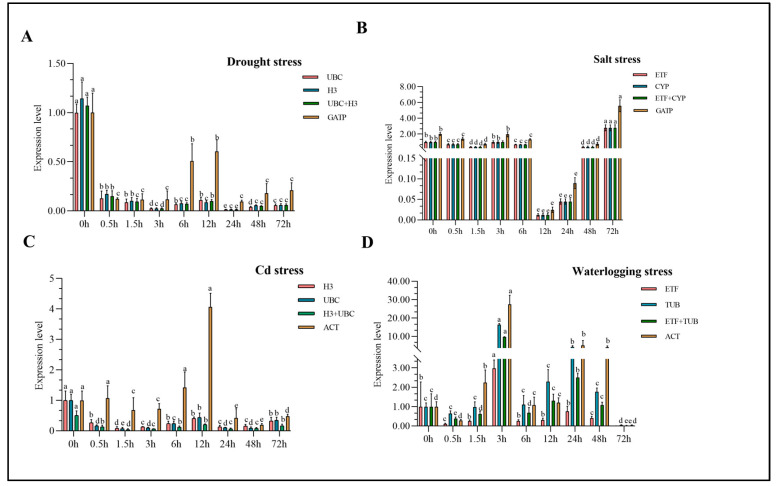
Expression levels of target genes in reed canary grass leaves under drought, salt, waterlogging and heavy metal stress. Different letters indicate significant differences in the expression of the same gene among different treatments (*P* < 0.05). (**A**) represents the expression of *PIP-1* in reed canary grass at different time points under drought conditions; (**B**) indicates the expression of *PAL* at different time points of salt stress in reed canary grass. (**C**,**D**) represent the expression of *NAC 90* and *WRKY 72A* in reed canary grass at different time points under waterlogging and heavy metal stress, respectively.

The stability of the candidate reference gene should be validated by selecting and normalizing the expression of the suitable target gene. In this study, target genes were chosen for each stress, and the two most and one least stable reference genes were selected for expression pattern analysis using the 2^−∆∆Ct^ method (Table 3). The most stable genes (*UBC* and *H3*) were suitable for normalizing *PIP-1* expression under drought stress, while the least stable gene (*GATP*) showed a different pattern (Figure 5A). Under salt stress, the selected most stable genes (*ETF + CYP*) normalized the expression of *PAL* genes compared to the least stable gene (*GATP*), and the normalization results showed different trends (Figure 5B). Similarly, under Cd stress, normalizing *NAC 90* using the unstable reference gene *ACT* resulted in a different trend than the most stable genes (*H3* and *UBC*) (Figure 5C). Under waterlogging stress, the most stable genes (*ETF* and *TUB*) exhibited similar trends, but the least stable reference gene (*TUB*) resulted in a different trend (Figure 5D). These results showed that the selected stable reference genes were reliable for this study, as they showed consistent expression patterns when used to normalize the expression of target genes under different abiotic stresses.

## 4. Discussion

Despite its important forage, bioenergy and ecological restoration functions, reed canary grass faces various complex stresses, such as drought, salt, Cd and waterlogging, which hinder its production applications. Therefore, it is urgent to study the response mechanism to the abiotic stress of reed canary grass. In particular, the accuracy of gene expression analysis is the basis and key linkage of abiotic stress research and analysis [25]. Although several studies have been conducted on the abiotic stress mechanisms in reed canary grass, there has been a lack of systematic comparative analysis regarding the stability of reference gene expression under abiotic stress conditions, and it could result in potential biases of the quantification of target gene expression in previous studies. Therefore, it is crucial to investigate the stability of reference gene expression under abiotic stress in reed canary grass to ensure accurate and reliable quantification of target gene expression in future studies [26,27,28].

Reference genes are typically expressed in metabolically active cells and perform essential cellular functions. They are important in cell cycle regulation [29,30,31]. The choice of reference genes and primer design have been reported to have a significant impact on the reliability of RT-qPCR target gene results [32,33]. In this study, the 13 candidate reference genes showed differential expression, consistent with previous studies reporting that reference genes are differentially expressed under different abiotic stresses of the same species. However, it is noteworthy that both *UBC* and *H3* performed well under drought and cadmium stress, which may indicate that *UBC* and *H3* genes can maintain stable expression regardless of abiotic stresses. The ubiquitin-coupled enzyme (E2), a driver of ubiquitin signaling in plants, has been reported to be highly tolerant to osmotic stress and cadmium stress by overexpression of ubiquitin-coupled enzyme (E2) [34,35,36]. In addition, this study found that the expression of *H3* varied with different treatments, and it suggested that *H3* may be a suitable reference gene under various abiotic stresses. However, *GATP*, a commonly used reference gene, was less stable than the others, verified in okra (*Abelmoschus esculentus*) [37]. In contrast, *TUB* was a more stable gene for expression under waterlogging stress, which was also demonstrated in *Betula alba* [38], and then *TUB* was the least stable in *Chrysoperla nipponensis* [39], okra and *Schima superba*, which also suggests that specific reference genes exhibit different expression stability depending on the species and stress they are exposed to.

Four main algorithms are commonly used to assess the stability of reference genes, including GeNorm, NormFinder, BestKeeper and RefFinder. GeNorm has different roles and purposes. GeNorm ranking is based on the similarity of expression levels of each reference gene from different experimental samples [22]. The stability of the candidate reference gene is calculated directly by BestKeeper using a pairwise correlation analysis of the Ct values [23]. To combine the results of the above three algorithms, an overall ranking was performed using the online ranking software RefFinder, which calculates the average gene weights and obtains the final ranking results [40]. RefFinder has now been applied to *Brassica juncea* [41], *okra* [38] and cucumber (*Cucumis sativus*) [42] for reference gene selection. GeNorm, NormFinder, BestKeeper and RefFinder were used in combination for gene expression analysis of candidate reference genes, but the differences in the first three algorithms resulted in inconsistent ranking of the stability of the reference genes analyzed by them. In this study, *H3* and *UBC* were the most stable reference genes when calculated by BestKeeper, and *UBC* and *H3* were also ranked first in terms of gene expression stability when calculated using GeNorm, but *UBC* was ranked second when calculated using the NormFinder algorithm, and the top-ranked reference gene was *ACT*.

*PIP-1*, *NAC 90*, *PAL* and *WRKY 72A* were targeted genes to validate the most and least stable reference genes selected under drought, salt, Cd, and waterlogging stresses. *PIP-1* is a water channel protein involved in drought stress in plants [43]. It has been reported that all 12 genes encoding water channel proteins (*PIP-1*, *PIP-2* and *NIP-2*) were significantly up-regulated under drought stress in *Miscanthus* [44]. In this study, *PIP-1* was selected as the target gene to validate a stable reference gene for drought stress, and the validation results showed that *PIP-1* exhibited a general expression pattern under drought stress, demonstrating the significance of the selected stable reference gene. The *PAL* is the first and key enzyme in the phenylpropane pathway and is transcriptionally regulated by various environmental factors. The *PAL* has been reported to be effectively applied at the molecular breeding level to improve the resistance of *Cyclocarya paliurus* under salt stress [45]. *PAL* was selected as a target gene to validate the reference gene for the stabilization of reed canary grass under salt stress, and the validation results were consistent with *PAL* expression levels under salt stress. *NAC 90* and *WRKY 72A* played different roles in different abiotic stresses and were selected as target genes to verify the stability of Cd stress and waterlogging stress, respectively [46]. This study selected *NAC 90* and *WRKY 72A* as target genes to verify the stable and stable reference genes for Cd stress and waterlogging stress, respectively.

## 5. Conclusions

This study provides a useful guideline for screening suitable reference genes to study the expression of reed canary grass genes under abiotic stress. The results showed that *UBC* and *H3* were more stable under drought and Cd stress; *ETF* and *CYT* were more desirable under salt stress; also, *ETF* and *TUB* were stable reference genes under waterlogging stress. These results provide an excellent functional genetic platform for studying reed molecular mechanisms under abiotic stress.

## Figures and Tables

**Table 1:** BestKeeper expression stability values for reed canary grass reference genes.

Rank	Drought	Salt	Cd	Waterlogging
1	H3 (0.87 ± 0.26)	CYT (1.4 ± 0.42)	H3 (1 ± 0.29)	H3 (1.26 ± 0.35)
2	UBC (0.94 ± 0.29)	ETF (1.42 ± 0.4)	UBC (1.22 ± 0.37)	ETF (1.27 ± 0.34)
3	MD (1.17 ± 0.36)	UBC (1.54 ± 0.46)	CYT (1.67 ± 0.51)	TUB (1.49 ± 0.4)
4	ACT (1.65 ± 0.49)	ACT (1.59 ± 0.44)	CUL1 (2.12 ± 0.52)	CUL1 (1.78 ± 0.44)
5	ETF (1.97 ± 0.55)	MD (1.73 ± 0.53)	TATA (2.2 ± 0.64)	UBC (2.13 ± 0.63)
6	CUL1 (2.05 ± 0.51)	TATA (1.83 ± 0.53)	GATP (2.39 ± 0.69)	50Sr (2.33 ± 0.57)
7	50Sr (2.65 ± 0.72)	Heats (1.85 ± 0.53)	Heats (2.48 ± 0.71)	ADPF (2.34 ± 0.69)
8	ADPF (2.66 ± 0.82)	TUB (2.02 ± 0.53)	MD (2.49 ± 0.76)	Heats (2.54 ± 0.74)
9	Heats (2.8 ± 0.85)	50Sr (2.06 ± 0.47)	50Sr (2.57 ± 0.64)	MD (2.73 ± 0.86)
10	CYT (2.95 ± 0.9)	ADPF (2.13 ± 0.63)	ADPF (2.89 ± 0.82)	GATP (3.14 ± 0.92)
11	TATA (3.38 ± 1)	H3 (2.66 ± 0.78)	ETF (3.01 ± 0.8)	CYT (4.35 ± 1.28)
12	TUB (3.59 ± 0.96)	CUL1 (2.69 ± 0.66)	TUB (3.01 ± 0.82)	TATA (4.4 ± 1.29)
13	GATP (4.1 ± 1.21)	GATP (3.3 ± 0.94)	ACT (4.26 ± 1.24)	ACT (4.48 ± 1.26)

**Table 2:** Expression stability values for candidate reference genes were calculated by NormFinder.

Rank	Drought	Salt	Cd	Waterlogging
1	ACT (0.353)	ETF (0.362)	H3 (0.436)	H3 (2.13 ± 0.63)
2	UBC (0.372)	CYT (0.382)	UBC (0.487)	ETF (3.14 ± 0.92)
3	H3 (0.421)	ETF (0.362)	50Sr (0.501)	TUB (1.27 ± 0.34)
4	MD (0.43)	CYT (0.382)	CYT (0.541)	UBC (1.49 ± 0.4)
5	CUL1 (0.614)	TUB (0.436)	TATA (0.566)	ADPF (4.35 ± 1.28)
6	ETF (0.749)	ACT (0.523)	CUL1 (0.666)	GATP (2.34 ± 0.69)
7	CYT (0.918)	UBC (0.584)	TUB (0.732)	Heats (1.26 ± 0.35)
8	50Sr (0.944)	MD (0.601)	Heats (0.768)	MD (2.33 ± 0.57)
9	Heats (0.958)	TATA (0.675)	GATP (0.794)	CUL1 (2.73 ± 0.86)
10	TATA (1.022)	ADPF (0.676)	ETF (0.833)	TATA (2.54 ± 0.74)
11	TUB (1.114)	50Sr (0.684)	MD (0.977)	CYT (1.78 ± 0.44)
12	ADPF (1.149)	CUL1 (0.763)	ADPF (1.098)	50Sr (4.4 ± 1.29)
13	GATP (1.288)	Heats (0.822)	ACT (1.141)	ACT (4.48 ± 1.26)

**Table 3:** The most stable and least stable reference gene combinations analyzed by RefFinder.

Method	Stability (High-Low)												
	1	2	3	4	5	6	7	8	9	10	11	12	13
	Drought												
BestKeeper	H3	UBC	MD	ACT	CUL1	ETF	50Sr	ADPF	Heats	CYT	TUB	TATA	GATP
Normfinder	ACT	UBC	H3	MD	CUL1	ETF	CYT	50Sr	Heats	TATA	TUB	ADPF	GATP
Genorm	UBC	H3	MD	CUL1	ACT	ETF	50Sr	CYT	Heats	TATA	TUB	ADPF	GATP
RefFinder	UBC	H3	ACT	MD	CUL1	ETF	50Sr	CYT	Heats	TATA	ADPF	TUB	GATP
	Salt												
BestKeeper	ETF	CYT	ACT	UBC	50Sr	TUB	TATA	MD	Heats	ADPF	CUL1	H3	GATP
Normfinder	ETF	CYT	TUB	ACT	UBC	MD	TATA	ADPF	50Sr	CUL1	Heats	H3	GATP
Genorm	ETF	TUB	CYT	ACT	UBC	MD	TATA	50Sr	ADPF	CUL1	Heats	H3	GATP
RefFinder	ETF	CYT	TUB	ACT	UBC	MD	TATA	50Sr	ADPF	CUL1	Heats	H3	GATP
	Cd												
BestKeeper	H3	UBC	CYT	CUL1	50Sr	TATA	GATP	Heats	MD	TUB	ADPF	ETF	ACT
Normfinder	H3	UBC	50Sr	CYT	TATA	CUL1	TUB	Heats	GATP	ETF	MD	ADPF	ACT
Genorm	UBC	H3	CYT	50Sr	TATA	CUL1	Heats	TUB	GATP	ETF	MD	ADPF	ACT
RefFinder	H3	UBC	CYT	50Sr	TATA	CUL1	Heats	TUB	GATP	MD	ETF	ADPF	ACT
	Waterlogging												
BestKeeper	ETF	H3	TUB	CUL1	50Sr	UBC	ADPF	Heats	MD	GATP	ACT	CYT	TATA
Normfinder	H3	ETF	TUB	UBC	ADPF	GATP	Heats	MD	CUL1	TATA	CYT	50Sr	ACT
Genorm	ETF	TUB	UBC	ADPF	H3	CUL1	GATP	Heats	MD	50Sr	CYT	TATA	ACT
RefFinder	ETF	TUB	H3	UBC	ADPF	CUL1	GATP	Heats	MD	50Sr	CYT	TATA	ACT

## Data Availability

Not applicable.

## Supplementary Materials

The supporting information can be downloaded at https://www.mdpi.com/article/10.3390/genes14091790/s1, Table S1. Sequence of 13 candidate reference gene. Table S2. 13 Candidate Reference Gene Primer. Table S3. Sequences of target genes under four abiotic stresses. Table S4. Primers for target genes under four abiotic stresses. Figure S1. Pearson’s correlation heat map based on four algorithms.
