# Supplementary figures and images for: Identification and Validation of Reference Genes for RT-qPCR Analysis in Reed Canary Grass during Abiotic Stress
Source: Genes (Basel). 2023 Sep 12;14(9):1790. doi: 10.3390/genes14091790 (PMC10530813; doi:10.3390/genes14091790)

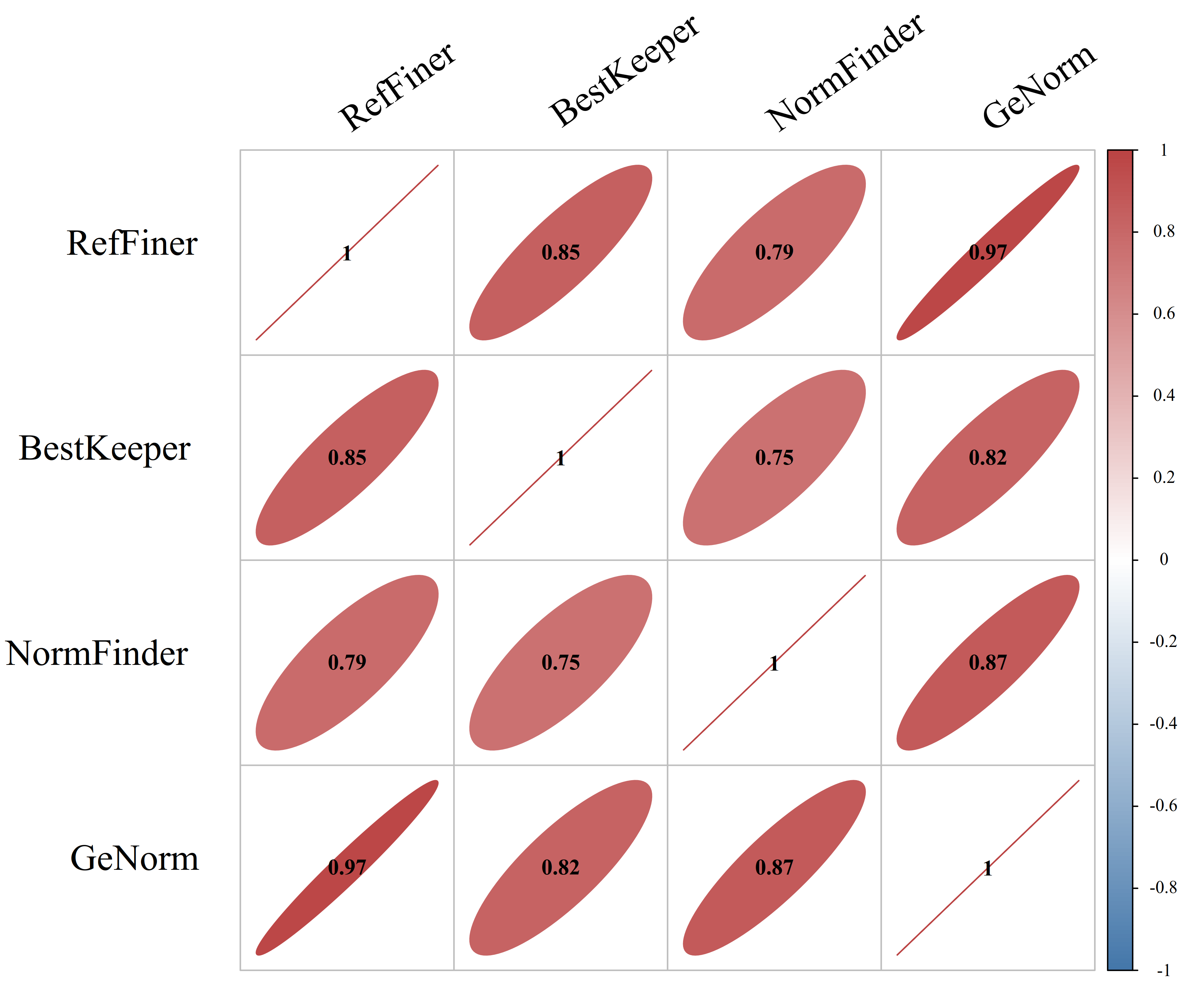

Supplement: Supplementary file 1 [file genes-14-01790-s001.zip › Figure S1.tif]
